# Elevated Soluble Fas and FasL in Cerebrospinal Fluid and Serum of Patients With Anti-N-methyl-D-aspartate Receptor Encephalitis

**DOI:** 10.3389/fneur.2018.00904

**Published:** 2018-10-25

**Authors:** Yue-Wen Ding, Su-Yue Pan, Wei Xie, Hai-Ying Shen, Hong-Hao Wang

**Affiliations:** ^1^Department of Neurology, Nanfang Hospital, Southern Medical University, Guangzhou, China; ^2^Department of Traditional Chinese medicine, Nanfang Hospital, Southern Medical University, Guangzhou, China; ^3^RS Dow Neurobiology Laboratories, Legacy Research Institute, Portland, OR, United States

**Keywords:** anti-NMDAR encephalitis, cerebrospinal fluid, Fas, Fas ligand, modified rankin scale

## Abstract

**Objective:** Anti-N-methyl-D-aspartate receptor (NMDAR) encephalitis is a severe autoimmune disorder that mainly affects children and young women. The Fas system contains both membrane-bound versions of Fas (mFas) and Fas ligand (mFasL), and soluble versions (sFas and sFasL), which play important roles in apoptosis and regulation of the immune system. Both the levels of sFas and sFasL and the role they play in anti-NMDAR disease pathogenesis remain unclear.

**Methods:** Forty-eight pairs of cerebrospinal fluid (CSF) and serum were collected from patients with anti-NMDAR encephalitis, encephalitis of other causes or peripheral neuropathy. The CSF and serum concentrations of sFas and sFasL were determined with enzyme-linked immunosorbent assay.

**Results:** CSF concentrations of sFas and sFasL were both increased in anti-NMDAR encephalitis patients compared with controls patients. Serum sFas levels were also elevated in anti-NMDAR encephalitis patients relative to controls. sFas and sFasL concentrations in CSF positively correlated with the modified Rankin scale (mRS) both at onset and 6-months follow-up.

**Conclusion:** CSF sFas and sFasL levels were elevated in anti-NMDAR encephalitis patients, and reflect the disease severity of anti-NMDAR encephalitis.

## Introduction

Anti-N-methyl-D-aspartate receptor (NMDAR) encephalitis is a newly recognized immune-mediated disorder that mainly affects children and young women (1). Clinical characteristics are associate with prominent memory deficit and psychiatric symptoms, seizures, movement disorder, autonomic instability or central hypoventilation (1, 2). Patients typically exhibit intrathecal synthesis of Antibodies against NR1 subunit of NMDAR (3, 4). Reports studying the effects of anti-NDMAR antibodies in the cerebrospinal fluid (CSF) of patients suggest an antibody mediated selective and reversible internalization of receptors from the cell surface (5, 6), leading to further pathogenesis (7).

Fas (CD95, apoptosis antigen 1) and Fas ligand (FasL, CD95L or TNFSF6) both belong to the death receptor subfamily of the TNF receptor superfamily (8). Extrinsic apoptosis pathway would be triggered upon Fas/FasL binding (9). Engagement of Fas induces oligomerization of preformed Fas trimers and recruits the Fas-associated death domain to form the death-inducing signaling complex (DISC). Upon activation, caspases in the DISC initiate the apoptotic signaling cascade (10).

The Fas/FasL complex play an important role in immune homeostasis by inducing cell apoptosis. In addition, recent data showed that Fas/FasL system also act as an effective chemoattractant for neutrophils, suggesting a potential pro-inflammatory function of these molecules (11). The Fas/FasL system contains both membrane-bound versions of (mFas and mFasL) and soluble versions (sFas and sFasL), sFas is mainly expressed on epithelial cells and activated lymphocytes, it regulates T cell homeostasis by mediates proliferation and death of T lymphocytes (12). High sFas levels have been associated with several autoimmune diseases including multiple sclerosis, autoimmune lymphoproliferation syndrome, and autoimmune thyroiditis (Hashimoto's thyroiditis) (13, 14). sFasL can be released from the cell surface by metalloproteinases, it retains the ability to bind Fas, but does not trigger apoptosis (15). sFasL expression on T cells has a reverse regulation effect, stimulating the maturation of antigen-stimulated T cells (9). Upon CNS injury, FasL overexpressed by neutrophils and macrophages, exerts chemoattractant to promote migration of these cells (16).

Though previous studies have confirmed the involvement of B and T cells in anti-NMDAR encephalitis, the immunopathogenesis and related apoptotic pathways have not yet been elucidated (4). In this study, we tested the CSF and serum sFas and sFasL levels in patients with anti-NMDAR encephalitis and analyzed the association of these proteins with disease outcome.

## Materials and method

### Patients

A total of 48 patients from the Neurology Department of Nanfang Hospital, Southern Medical University were enrolled. Twenty-four patients with anti-NMDAR encephalitis were diagnosed according to published criteria (17). Twenty-four patients with non-inflammatory neurologic disorders were selected as controls. Among these controls, 13 patients were diagnosed with encephalitis of other causes (EOC) (seven with demyelinating disease, three with viral encephalitis and three of unknown cause) (Supplementary Table 1) and 11 patients were diagnosed with peripheral neuropathy (PN). There was no significant difference in age and gender between the groups. All patient serum and CSF samples were tested for antibodies against the NR1 subunit of NMDAR using cell-based analysis (FA 112d-51, Oumeng, Beijing).

Neurological status was assessed using the modified Rankin Scale (mRS) at the most critical time of disease and 6 months after symptom onset. The study protocol was approved by the ethics committee of the Nanfang Hospital, Southern Medical University, and written informed consent was obtained from each participant.

### Sample collection

CSF and serum samples were obtained from patients within 3 days of admission during routine clinical practice. CSF samples were collected using polypropylene tubes and centrifuged at 4,000 g for 10 min. Blood samples were collected using Vacutainer Serum Separation tubes and allowed to clot for 20 min at room temperature. Serum was separated by centrifugation at 1,000 g for 10 min and transferred into a new tube prior to storage. All samples were stored at −80°C within 60 min of collection.

### Enzyme-linked immunosorbent assay

Human sFas and sFasL concentrations in CSF and serum samples were determined using commercially enzyme-linked immunosorbent assay (ELISA) kits (CSB-E04542h, CSB-E04544h, Cusabio Biotech, Wuhan). CSF samples were assayed undiluted and serum samples were diluted 1:2 prior to use in the assay. Sandwich ELISA was performed according to the manufacturer's instructions. All samples were measured in duplicate.

### Statistical analysis

All data were statistically analyzed using SPSS 20.0. Data were expressed as mean ± SEM. Differences in levels Fas and FasL between the anti-NMDAR encephalitis and controls group were analyzed using the one-way ANOVA test. The correlation statistics regarding mRS scores were calculated by Spearman test. All graphics were generated with GraphPad Prism 7*. p-*values: ^*^ < 0.05; ^**^ < 0.01; ^***^ < 0.001

## Results

### Clinical characteristics of recruited anti-NMDAR patients

The clinical characteristics of recruited patients are summarized in Table 1. In this anti-NMDAR encephalitis patients' cohort, 11 (45.8%) had prodromal symptoms (headache, fever), 23 (95.8%) had psychiatric symptoms or memory deficiency, 11 (45.8%) had seizures, 13 (54.2%) had autonomic symptoms, 15 (62.5%) had consciousness disturbance, 14 (58.3%) had movement disorder, and three (12.5%) had ovarian teratoma. The mean onset mRS was 3.75 ± 0.79, which was significantly different from the mRS at 6-months follow-up (2.38 ± 0.97). All patients were identified with positive anti-NMDAR antibody in CSF and 18 (75%) patients with positive serum anti-NMDAR antibody.

**Table 1:** Clinical characteristics of patients.

	**Anti-NMDAR encephalitis**	**Encephalitis of other causes**	**Peripheral neuropathy**
Number of patients (*n*)	24	13	11
Gender (female/male)	14/10	8/5	5/6
Age (years)	39.58 ± 17.51	39 ± 18.93	45.64 ± 13.82
**Number of symptoms (*****n*****)**			
Prodromal symptoms (headache, fever)	11	4	0
Disorders of memory, behavior, and cognition	23	3	1
Seizures	11	1	0
Autonomic symptoms	13	2	1
Loss of consciousness	15	4	0
Movement disorder	14	7	9
Ovarian teratoma	3	0	0
Maximum mRS	3.75 ± 0.79	2.92 ± 1.55	
6 months' mRS	2.38 ± 0.97	1.85 ± 1.21	
CSF anti-NMDAR antibody	24	0	0
Serum anti-NMDAR antibody	18	0	0

### sFas and sFasL concentration are increased in anti-NMDAR encephalitis patients

**Figure 1:**
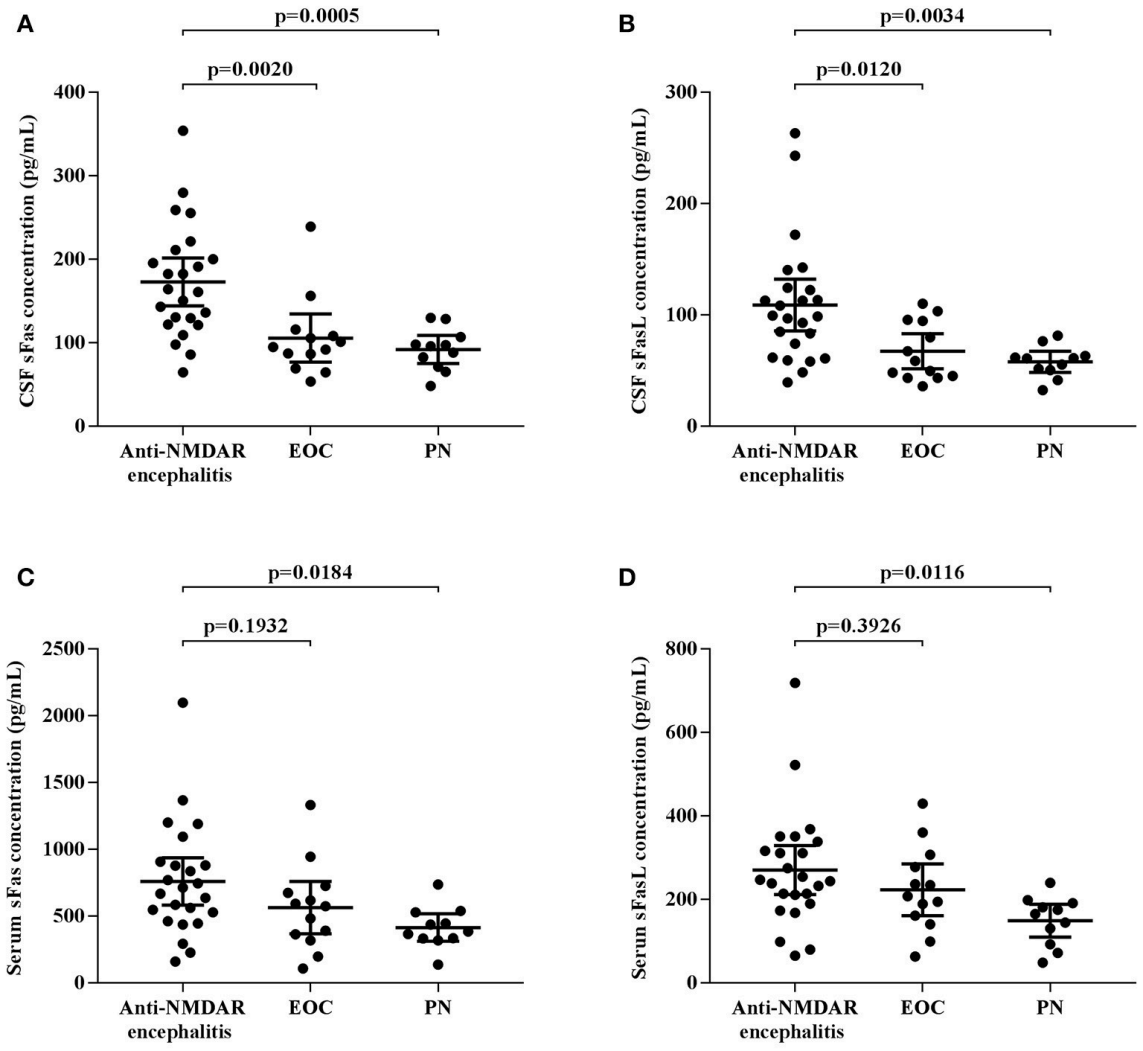
CSF and Serum concentration of sFas/sFasL in anti-NMDAR encephalitis patients and controls **(A–D)** (one-way ANOVA).

**Figure 2:**
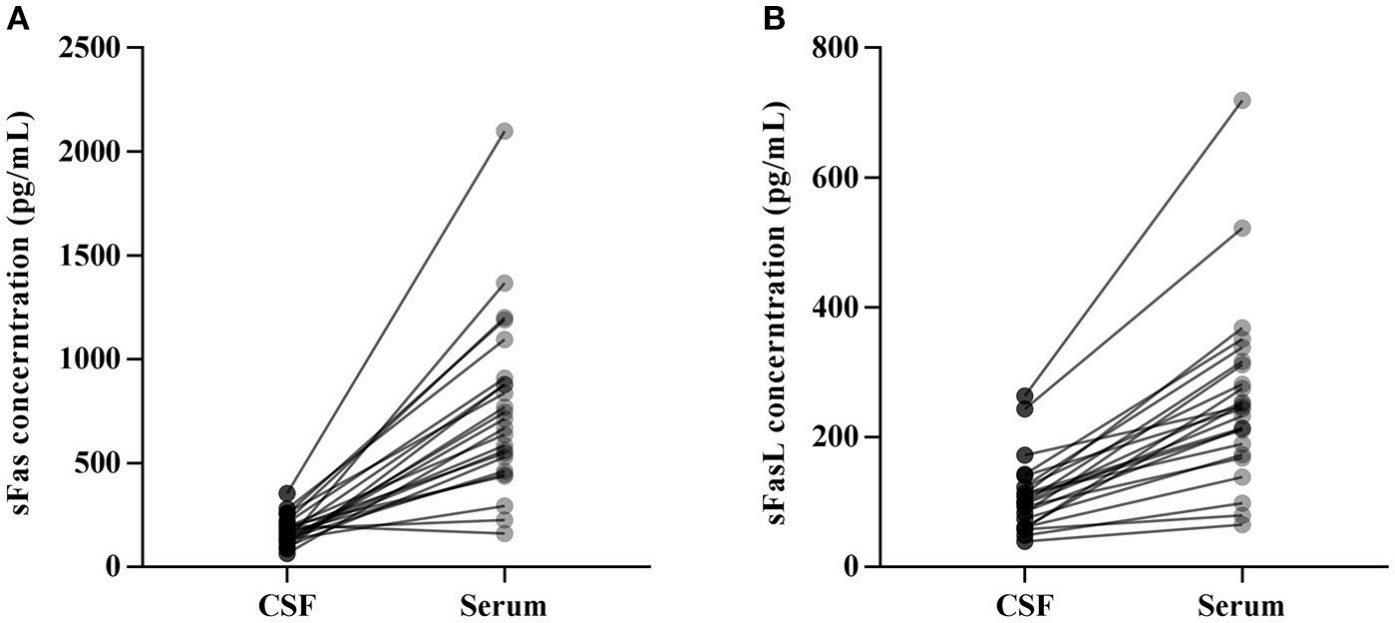
Concentration of CSF and Serum sFas/sFasL in anti-NMDAR encephalitis patients **(A, B)**.

CSF and serum concentrations of sFas and sFasL are presented in Figure 1. Patients with anti-NMDAR encephalitis had higher CSF levels of sFas and sFasL than those in the control groups (sFas in CSF: *p* < 0.01 [anti-NDMAR vs. EOC], *p* < 0.001 [anti-NDMAR vs. PN]; sFasL in CSF: *p* < 0.05 [anti-NDMAR vs. EOC], *p* < 0.01[anti-NDMAR vs. PN]). Serum concentrations of sFas were higher in anti-NMDAR encephalitis patients than in patients with PN (*p* < 0.05). No significant difference was observed between the sFasL level in anti-NMDAR encephalitis and EOC groups. As shown in Figure 2, serum levels of sFas and sFasL were remarkably higher than those in the CSF (sFas: 140.9 ± 69 pg/mL, *p* < 0.001 [serum vs. CSF]; sFasL: 515 ± 396.3 pg/mL, *p* < 0.001 [serum vs. CSF]). Furthermore, the receiver operating characteristic (ROC) analysis was used to evaluate the diagnostic potential of sFas and sFasL for anti-NMDAR encephalitis (Table 2). In the comparison of patients with anti-NMDAR encephalitis and non-anti-NMDAR encephalitis, the area under the ROC curve (AUC) was 0.8585 for CSF sFas, which was superior to CSF sFasL (AUC: 0.8056) and serum sFas and sFasL (AUC: 0.7222, 0.6858, respectively). The optimal cut-off values for CSF and serum sFas and sFasL levels were: 121.2, 83.5 pg/mL (CSF sFas, sFasL, respectively) and 280.5, 106.7 pg/mL (serum sFas, sFasL, respectively), both CSF and Serum of sFas/sFasL are showed improved diagnostic accuracy when differentiating patients with Anti-NMDAR encephalitis from those with PN.

**Table 2:** ROC analysis of CSF and Serum sFas/sFasL.

	**AUC**	**95% CI**
**ANTI-NMDAR ENCEPHALITIS VS. NON-ANTI-NMDAR ENCEPHALITIS**		
CSF sFas	0.859	0.745–0.972
CSF sFasL	0.806	0.681–0.930
Serum sFas	0.722	0.575–0.869
Serum sFasL	0.686	0.566–0.864
**ANTI-NMDAR ENCEPHALITIS VS. EOC**		
CSF sFas	0.829	0.679–0.979
CSF sFasL	0.779	0.628–0.930
Serum sFas	0.644	0.456–0.832
Serum sFasL	0.619	0.425–0.812
**ANTI-NMDAR ENCEPHALITIS VS. PN**		
CSF sFas	0.894	0.788–0.999
CSF sFasL	0.837	0.707–0.968
Serum sFas	0.814	0.670–0.959
Serum sFasL	0.830	0.695–0.964

*CSF, cerebrospinal fluid; AUC, area under the curve; EOC, Encephalitis of other causes; PN, Peripheral neuropathy*.

### Increased CSF sFas and sFasL levels correlated with clinical outcome and follow-up

**Figure 3:**
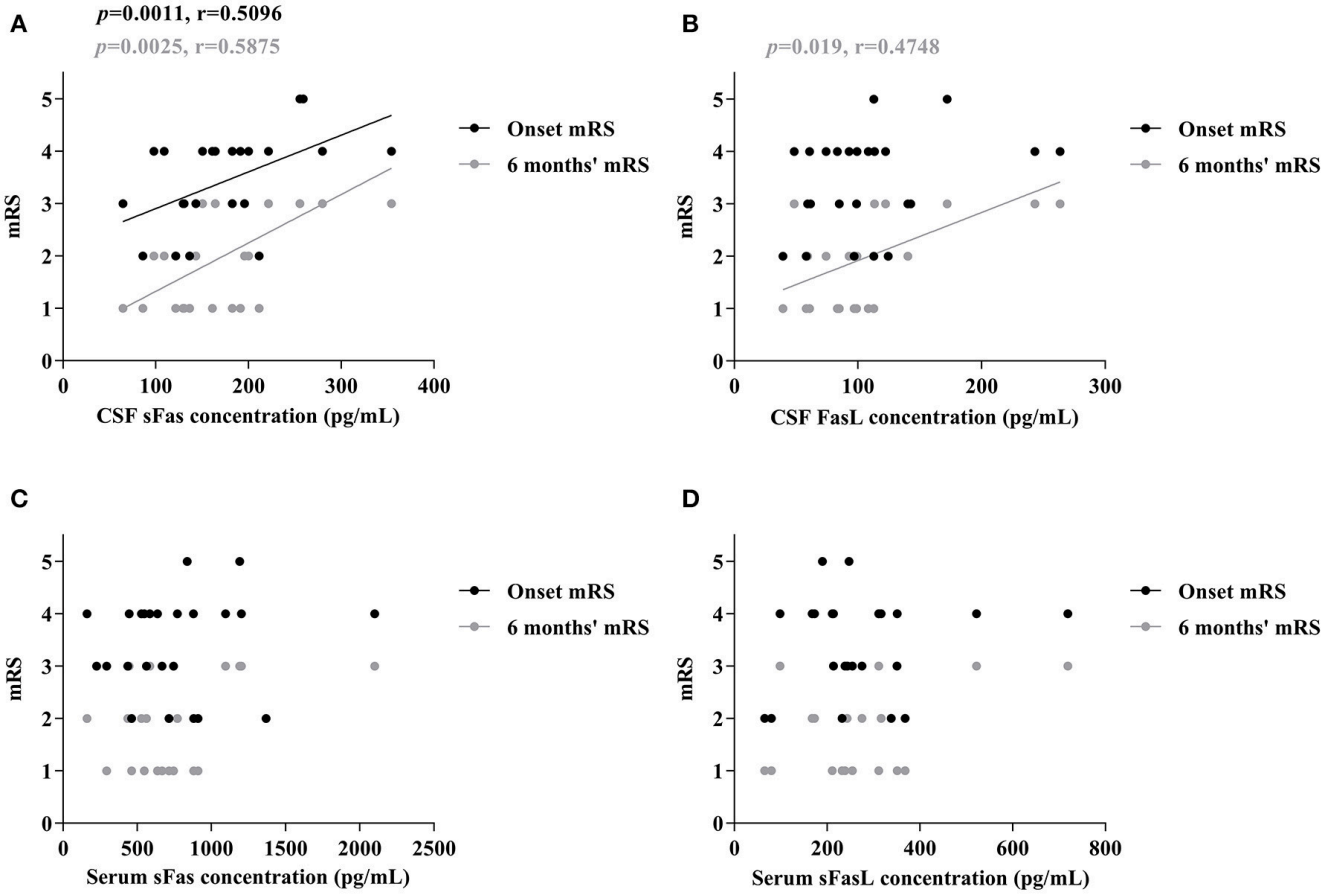
Correlation of CSF and Serum sFas/sFasL levels with max mRS and 6 months' mRS in anti-NMDAR encephalitis patients **(A–D)** (Spearman test).

To assess whether sFas and sFasL levels are associated with disease severity and progression, mRS scores were evaluated at the most critical time and 6 months after symptom onset. As shown in Figure 3, increased CSF sFas level was significant correlated with disease severity (*p* = 0.0011). Both sFas and sFasL levels in CSF correlated with disease severity at the 6-months follow-up (*p* = 0.0025 [sFas], *p* = 0.019 [sFasL]), but serum levels of sFas and sFasL did not correlate with onset mRS or mRS at 6-months follow-up.

## Discussion

In this case–control study, we examined the concentrations of sFas and sFasL in 48 pairs of CSF/serum samples from patients with anti-NMDAR encephalitis (18) and controls (18). The results showed increased intrathecal levels of sFas and sFasL in anti-NMDAR encephalitis patients, which significantly correlated with both onset mRS and mRS at 6-months follow-up.

In the CNS, sFas is expressed in neurons, astrocytes and Lymphocyte (19, 20). The role of sFas was confirmed in several neurological diseases, including multiple sclerosis, Alzheimer's disease and hydrocephalus and further identified as a diagnostic marker of these disease (21–23). The apoptosis of neuronal cells may also be involved in the course of anti-NMDAR encephalitis. Studies confirmed that blockade of the NMDAR led to apoptosis and neurodegeneration in the neonatal rodent brain (24). The increase in extracellular glutamate and excessive calcium influx may affect neuron survival (6).

In addition to apoptotic effects, researchers have also demonstrated the immune-regulatory function of Fas/FasL signaling. mFasL can be cleaved by different metalloproteinases to produce sFasL which is released into the extracellular environment (18). Although sFasL can interact with Fas, it does not trigger the progression of cell death (25). In contrast, it inhibits the interaction between Fas and FasL on the cell surface and blocks cell death (23). Studies have shown that in various cell lines, sFasL binding to Fas can induce cell proliferation but does not induce apoptosis; it can also trigger the accumulation of certain T cell subsets in damaged organs (26). The overexpression of sFasL in the serum of patients has been observed in both SLE and breast cancer and is reported to contribute to disease severity (27, 28).

The pathogenicity of B cells in anti-NMDAR encephalitis has been confirmed (4, 29). The beneficial effect of plasma exchange also indicate the relevance of humoral immune response in anti-NMDAR encephalitis (17). Though most patients respond to immunotherapy, anti-NMDAR antibodies can be detected in the CSF 6 months after immunotherapy initiation (1, 7), which may attribute to the persistent intrathecal synthesis of antibodies and the presence of brain-infiltrating plasma cells (30).

Studies have shown that Fas/FasL signaling in B cells contributes to cellular processes such as maturation, proliferation and immunoglobulin production (11). Fas is highly expressed in activated B cells (31), the dysfunction B cell-specific Fas is associated with the onset of autoimmunity (32). In anti-NMDAR encephalitis patients, overexpression of sFas and sFasL may block the Fas/FasL interactions on the cell surface, thus preventing the apoptosis of B cells induced by Fas/FasL signaling.

Early treatment is tightly linked to the prognosis of anti-NMDAR encephalitis, a sensitive biomarker may assist in the treatment and follow-up of these patients. In the present study, we found that the CSF levels of sFasL were positively correlated with both the onset mRS scores and mRS scores at 6-months follow-up, indicating that higher levels of CSF sFasL are associated with a more severe clinical presentation and a worse prognosis. Therefore, CSF sFas levels, combined with anti-NMDAR antibody titer change, may be a promising biomarker for monitoring patients with anti-NMDAR encephalitis.

There are several limitations to the present study. First, this study investigated a relatively small number of patients, due to the lack of following up data, it's hard to evaluate the relevance of sFas and sFasL in disease prognosis. In addition, sFas and sFasL is not disease specific and may involved in other neurological conditions like neurodegenerative disease and infectious diseases. Finally, this was just a preliminary study, the mechanism of the increases sFas and sFasL in anti-NMDAR encephalitis patients remain unclear, which should be elucidated with further studies.

## Conclusion

Our study indicated that CSF and serum levels of sFas and sFasL are increased in anti-NMDAR encephalitis patients and may be a sensitive marker for disease severity. Future studies are required to elucidate the physiological relevance of high sFas and sFasL levels in anti-NMDAR encephalitis described herein.

## Author contributions

H-HW, H-YS, and WX co-conceived this study and designed the experiments. Y-WD, S-YP, WX, and H-HW collected the CSF samples and clinical data. Y-WD and H-HW performed the experiments and analyzed the data. Y-WD, H-HW, and H-YS wrote the manuscript and prepared the table/figures. All authors read and approved the final manuscript and agreed to submit it for publication.

### Conflict of interest statement

The authors declare that the research was conducted in the absence of any commercial or financial relationships that could be construed as a potential conflict of interest.
