# Supplementary material for: Elevated Soluble Fas and FasL in Cerebrospinal Fluid and Serum of Patients With Anti-N-methyl-D-aspartate Receptor Encephalitis
Source: Front Neurol. 2018 Oct 25;9:904. doi: 10.3389/fneur.2018.00904 (PMC6209679; doi:10.3389/fneur.2018.00904)
Supplement: Supplementary file 1 [file Table_1.DOCX]

**Supplementary Table 1.** The clinic manifestations and diagnosis of patients with encephalitis of other causes

| Gender | Age (years) | Clinical symptoms | Days of hospitalization | Diagnosis |
| --- | --- | --- | --- | --- |
| Male | 34 | Movement disorder, Impaired vision | 14 | Neuromyelitis optica |
| Male | 49 | Headache, fever, movement disorder, dysphagia | 25 | Unknown aetiology |
| Male | 15 | Headache, fever | 19 | Herpes simplex virus encephalitis |
| Male | 12 | Visual impairment, dizzy | 34 | Unknown aetiology |
| Female | 73 | Movement disorder, dysphagia | 22 | Neuromyelitis optica |
| Male | 45 | Movement disorder | 14 | Multiple sclerosis |
| Male | 59 | Visual impairment, vomit | 9 | Neuromyelitis optica |
| Female | 25 | Movement disorder, visual impairment | 18 | Neuromyelitis optica |
| Female | 53 | Movement disorder, visual impairment | 11 | Neuromyelitis optica |
| Male | 11 | Headache, fever, visual impairment | 23 | Varicella zoster virus encephalitis |
| Female | 59 | Movement disorder | 15 | Neuromyelitis optica |
| Female | 22 | Headache, fever, seizure | 17 | Herpes simplex virus encephalitis |
| Male | 47 | Dizzy, Seizure | 30 | Unknown aetiology |
